# Knowledge, attitude, and perceptions of healthcare professionals towards complementary and alternative medicine: a cross-sectional survey from twin cities of Pakistan

**DOI:** 10.1186/s12906-023-04187-2

**Published:** 2023-12-01

**Authors:** Azhar Hussain Tahir, Maria Tanveer, Gul Shahnaz, Muhammad Saqlain, Shagufta Ayub, Ali Ahmed

**Affiliations:** 1 District Headquarter Hospital, Khushab, 41000 Punjab Pakistan; 2 Department of Pharmacy, Quaid-I-Azam University, Islamabad, 45320 Pakistan; 3 Leads College of Pharmacy, Lahore, 54000 Punjab Pakistan; 4 Riphah Institute of Pharmaceutical Sciences, Riphah International University, Islamabad, Pakistan

**Keywords:** Complementary and alternative medicine, Pakistan, Attitude, Knowledge, Perception, Healthcare professionals

## Abstract

**Background:**

The growing popularity and use of complementary and alternative medicine (CAM) products among the general public worldwide has been well documented. This study aimed to investigate the knowledge, attitude, and perceptions (KAP) of Pakistani healthcare professionals (HCP) toward CAM and to document their views on integrating CAM education with the curriculum of undergraduate health science programs.

**Method:**

A cross-sectional study using simple random sampling was conducted for a duration of ten months among HCPs from Pakistan’s twin cities: Islamabad and RawalpindiThe data were collected using a self-administered and validated (Cronbach’s alpha: 0.71) questionnaire. This questionnaire consisted of five sections, namely; demographic, attitude, perception, integration and knowledge.

**Result:**

The response rate was 91.20% (500/456). The participants included 160 physicians, 155 nurses,and 141 pharmacists. The majority of the respondents were females, 67.50%, and unmarried (60.50%).The majority of HCPs participating in this study agreed that CAM modalities may benefit conventional medicine system. Likewise, most HCPs perceived different CAM therapies aseffective treatment options. More than 50% HCPs suggested CAM elective courses in the curriculum of the health sciences program.Overall, 79.17% of the HCPs have poor knowledge of CAM. Physicians have the highest knowledge score 25.63%, followed by pharmacists 21.99%, and nurses 12.26%. Knowledge status was significantly associated with age, profession, and experience of practice (p = 0.001,0.001 & 0.019).

**Conclusion:**

This study revealed that despite the overall positive attitude of HCPs toward CAM, the score of knowledge is low. Therefore, the survey recommends evidence-based guidelines for the rationale use of CAM and updated syllabi of undergraduate health programs which will assist the future HCPs in increasing professionals’ knowledge toachieve better health outcomes for the general public.

## Background

The World Health Organization (WHO) defines CAM) as “a broad set of healthcare practices that are not part of that country’s tradition and are not integrated into the dominant healthcare system” [1, 2].These practices are defined as “alternative” when they are used instead of allopathic medicine and as “complementary” when they are used together with it [3]. Herbal medicine (Unani medicine & Ayurveda Medicine system), homeopathy, and acupuncture are among the acceptable CAM modalities in the world [4, 5]. There are five main types of CAM using all over the world: whole body systems (unani medicine, homeopathic medicine, traditional Chinese medicine, Ayurveda medicine etc), Mind body system (Spiritual healing, prayers etc.), Biologically based therapies (dietary supplement, vitamins, foods, herbs etc.),Manipulative and body-based therapies (Massage), Energy medicine systems (Reiki) [6]. WHO provides supportto member countries through technical guidance and formulating strategiesto promote the safe use of CAM, to prevent and cure diseases. Regulating CAM usage is of paramount importance, especially for low or middle-income countries (LMIC),considering that approximately 80% of the population in developing countries use traditional / alternative medicine as primary health care [7–9]. Scientific literature shows that in developing countries many patients with chronic diseases are reliant on CAM [10, 11]. CAM are used for treatment of different chronic conditions such as Metabolic Syndrome (MetS) and its components such as diabetes, hypertension, dyslipidaemia and central obesity [12].According to a study conducted in Iran, almost 72.4% of hypertensive patient used CAM [13]. Different herbs are used for anti-inflammatory and anti-ulcergenic action [12]. In a randomized clinical trial, medicinal plant known as Malva sylvestris L. (M. sylvestris) was used for the treatment of atopic dermatitis in children and it gave fruitful results [12–14].Due to the accessibility and escalating cost of healthcare services coupled with growing knowledgeabout natural products and their usage in the treatment of diseases. Patients’ interest in CAM has increased significantly over the past few years in both developed and developing countries [8, 15, 16].

Pakistan has a long history of traditional and alternative medicine where numerous CAM healers provide services as Hakims/Tabibs (unani medicine healers),homeopathic doctors and spiritual healers [17]. In Pakistan, the National Council for Tibb (NCT) and National Council for Homeopathy (NCH)regulate the practice of CAM under the supervision of the Ministry of National Health Services Regulation and Coordination Islamabad (NHSR&C). Drug Regulatory Authority of Pakistan (DRAP)is responsible for the proper enlisting of CAM products in Pakistan [18, 19]. In Pakistan, 40–59% of the population uses CAM for various health issues [2, 7]. Increasing community willingness to use CAMs necessitates healthcare professionals’ knowledge of various CAM modalities; they can warn patients about these treatments’ risks and side effects and respond to their questions [19].Being an umbrella of the healthcare system, the role of Physicians in guiding patients tosafe and efficacious use of CAM practices is second to none [20]. Pharmacists and nurses are key healthcare workers; therefore, they have an essential role in appropriately using CAM in healthcare by providing correct information [21–23]. Therefore, healthcare policymakers have been focusing on the integration of CAM into the curriculum of health sciences courses [24].

Concomitant use of alternative and conventional medicines is common in Pakistan [17] and belief on faith healers for treatment of chronic disorders like epilepsy is prevalent [25, 26], making it even more important to disseminate accurate knowledge. Given that alternative medicine can interact with or reduce the therapeutic effects of allopathic medicine, HCPs with current knowledge of CAM are needed to guide patients and avoid adverse reactions [27]. Knowledge, attitude, and perception (KAP) surveys are commonly used in public health research to assess KAP about a particular health issue or behavior [28–32]. By collecting this information about KAP, policy makers and public health professionals can identify knowledge gaps and behaviour patterns specific to the Pakistani population, which can help them design and implement effective healthcare interventions and enforce targeted public health policies.Therefore,this study aims to explore the KAP of Pakistani HCPs toward CAM. A secondary objective of the study was to collect the opinions of HCPs about integrating CAM courses intothe undergraduate curriculum of health science programs.

## Methods

### Study design and settings

A multi-center cross-sectional survey was carried out in Islamabad and Rawalpindi, the twin cities of Pakistan, from March 2022 to December 2022. Data was collected using a validated questionnaire to target HCPs of five specialized care government hospitals; polyclinic hospital, Islamabad, Pakistan Institute of medical sciences (PIMS), Islamabad; Benazir Bhutto Hospital (BBH), Rawalpindi; District Head Quarter (DHQ) hospital, Rawalpindi, Holy family hospital, Rawalpindi, two private secondary care hospitals (Maroof International hospital, Islamabad & Ali medical complex, Islamabad) and four chain pharmacies (D. Watson, Shaheen, Latif chemist, Ahmed chemist).

#### Study population

The study participants were physicians, pharmacists, and nurses of selected hospitals and pharmacies. HCPs other than physicians, pharmacists, and nurses like dentists, physiotherapists, and medical lab technologists were excluded. Furthermore, those who refused to participate and healthcare practitioners of alternative medicine systems were also excluded.

### Measurement tool and its validity

The authors developed a 45 items questionnaire after in-depth literature review [22, 24, 33, 34] and visiting NCT & NCH under the control of the Ministry ofNHSR&C, Islamabad to check the CAM work nature in Pakistan. This questionnaire was consisted of five sections namely; demographic, attitude, perception, integration and Knowledge. A primary description of this tool was sent to senior pharmacy academics/experts and HCPs, including practitioners of herbal & homeopathic medicine, for content validation and relevance. Their assessment was integrated to adjust our measurement tool according to study objectives. A pilot study was carried out on easily available 45 HCPs, including an equal number of physicians, nurses, and pharmacists, for face validation of study tools who were later excluded from the final analysis. The internal consistency of the questionnaire was checked by SPSS Version 26, and the overall value of Cronbach’s alpha was found to be 0.71, which was considered reliable. The Cronbach alpha values for attitude, perception, integration and knowledge sections were 0.71, 0.72, 0.72 and 0.70, respectively.

### Sample size and sampling technique

The sample size calculated by Cochran Eq. (1977) (n= (z (α/2)) 2 p (1 − p)/d2) of sampling was 384, taking 0.05 margin of error at 95% confidence level [35]. P = proportion of HCPs who practice CAM. Participants were selected by using a convenience sampling technique. AHT, and AA, distributed questionnaires with consent forms among the selected participants and also briefed about them aboutstudy aims and objectives and assured them of their secretiveness. For a possible nonresponse rate, 10% of the total sample size was considered, and made a final sample size of 422. We enrolled 500 HCPs of selected hospitals and pharmacies to avoid errors and get more reliable results. Out of 500 questionnaires distributed to consented participants, 456 (91.2%) questionnaires were returned (160 Physicians, 141 Pharmacists, 155 nurses), so the response rate was 91.2%.

### Statistical analysis

All information collected through formulated questionnaire was coded into variables. Except forthe name, the demographic profile of study contributors was entered as categorical variables. Knowledge, attitude, opinion on the integration, and perception of study contributors were added as continuous variables. A 5-point likert scale was used for the attitude, perception and integration sections. In the attitude and integration sections, responses of agree and strongly agree were consideredas positiveresponses. While answers given by using disagree and strongly disagree ere considered negative responses. Neutral answers are dealt separately as neutral responses.Similarlyperception about different CAM modalities was assessed after collecting the answers on likert scale. Answers given by choosing “effective” and “very effective”were counted as vote for the effectiveness of mentioned CAM modality while answers of “harmful” and “very harmful” options were calculated as a vote for harmfulness. The knowledge of those participants was considered as good, who gave 10 or more correct answers, while the knowledge of those participants considered as poor, who gave less than 10 correct answers.

- **Categorical variables** This part includes questions such as marital status, gender, age, city of practice, area (rural and urban), profession, experience, and category of practice.
- **Continues variables**.

1. **Attitude section**: second part assessed the attitude of participants and had ten questions, and each item was secured on a 5-point Likert scale and measured individual’s attitudes toward CAM therapies.
2. **Perception section**: In this section, fivequestions about the effectiveness and harmfulness of five different CAM modalities were asked from participants and assessed on a 5-point Likert scale.
3. **Integration section**: To check the opinion and attitude toward statements about the integration of CAM education in the curriculum of health science courses, six questions were asked from participants and assessed on a 5-point Likert scale. Responses to statement no.6 were reverse-coded because this was a negative narrative statement.
4. **Knowledge section**: this section consisted of 16-item knowledge test questions on indications of CAM products and basic concepts about different CAM modalities intended to check the HCP’s knowledge.

After coding of collected data, inferential and descriptive statistical analysis was conducted using SPSS version 26 for analysis of data. Descriptive statistics were applied to estimate the frequencies and percentages. The association of dependent variables (knowledge, attitude, opinion about integration of CAM and perception) and independent variables (demographics items) were estimated using Pearson’s Chi-square.

### Ethical considerations

The study was performed in accordance with the declaration of Helsinki [36] and after securing ethical permission from the Ethical Review Board of Shaheed Zulfiqar Ali Bhutto Medical University (Letter No: F.1–1/2022/ERB/ SZABMU/0/121). Furthermore, informed consent was taken from each study participant before collecting the data. The responses of participants were kept confidential.

## Results

### Demographic characteristics

Most of the HCPs were females (308, 67.50%), unmarried (276, 60.50%), urban residents (322, 70.61%), and fromthe age group 20–30 years (293,64.25%). When comparing the different professions, physicians were more in number (160, 35.09%) than others, followed by nurses (155,33.99%) and pharmacists (141 30.92%).Responses to demographic questions are shown in Table 1.

**Table 1:** Demographic characteristics of study population

Demographic variables	Frequency (%)
**Age (years)**	
20–30	293(64.25)
31–40	120(26.32)
41 and above	43(9.43)
**Profession**	
Physician	160(35.09)
Pharmacist	141(30.92)
Nurse	155(33.99)
**Gender**	
Female	308(67.5)
Male	148(32.5)
**Experience of practice (in years)**	
0–3	272(59.65)
4–6	103(22.59)
7–9	53(11.62)
10 and above	28(6.14)
**Category of Practice**	
Secondary	211(46.27)
Tertiary	245(53.73)
**Area of living**	
Rural	134(29.39)
Urban	322 (70.61)
**Marital status**	
Yes	180(39.5)
No	276(60.5)
**City**	
Islamabad	278(60.96)
Rawalpindi	178(39.04)

### Attitude towards CAM

Most of the participants showed a positive attitude toward all statements except one statement, “clinical care should integrate conventional medicine and CAM” where 42.32% (193) of participants showed a negative attitude with a significantdifference by profession (p = 0.02) and category of practice (p = 0.04). 41.88% (130) of participants agreed that CAM includes the ideas and methods from which conventional medicine could benefit. This response significantly differed by the experience of practice (p = 0.011) and marital status (p = 0.02). Similarly, 52.41% (239) of participants respond positively to the statement “Knowledge of CAM is important to me as a health care professional” with a significant difference by category of practice (p = 0.02). Likewise, 43.2% (197) of participants agreed that health professionals should be able to advise patients on the commonly used CAM method with a significant difference by profession (p < 0.001), Gender (p = 0.047), the experience of practice (p = 0.012) and category of practice (p = 0.046). 45.39% (207) of respondents believed that CAMs offer cost-effective treatment options with a significant difference by profession (p = 0.002) and marital status (p = 0.049). Regarding the statement “CAMs need more hospital-based research” about 53.29% (243) of participants agreed. The response to this statement was significantly different among professionals (p = 0.04). Almost 53% (241) of participants showed their willingness for scientific testing of CAM with a significant difference by profession (p = 0.049), experience of practice (p = 0.041), category of practice (p = 0.02), and marital status (p = 0.04). Percentage values of positive and negative attitudes and their respective p-values for all demographic variables can be seen in Table 2.

**Table 2:** Attitude of HCPs toward CAM and differences in response by demographic characteristics

Questions about attitudes	SD	D	N	A	SA	A	P	G	EP	AL	CP	MS	R
Clinical care should integrate conventional medicine and CAM	82 (17.98)	111 (24.34)	88(19.29)	124(27.19)	51(11.18)	0.4	**0.02**	0.263	0.111	0.258	**0.04**	0.08	0.09
CAM includes the ideas and methods from which conventional medicine could benefit	35 (7.68)	95(20.83)	135(29.61)	152(33.33)	39(8.55)	0.08	0.06	0.48	**0.011**	0.101	0.223	**0.02**	0.39
Treatment not tested in scientific manners should be discouraged.	36 (7.90)	56(12.28)	104(22.81)	153(33.55)	107(23.46)	0.21	**0.03**	0.17	0.53	0.135	**0.02**	0.136	0.18
Health and disease are the balance between enhancing and destructive forces.	38 (8.33)	93(20.40)	109(23.90)	164(35.97)	52(11.40)	0.19	**< 0.001**	0.06	0.171	0.39	**< 0.001**	0.25	0.253
Knowledge of CAM is important to me as a healthcare professional.	42 (9.21)	47(10.31)	128(28.07)	164(35.96)	75(16.45)	0.40	0.174	0.29	0.21	0.165	**0.002**	0.28	0.175
Health professionals should be able to advise patients on the commonly used CAM method.	35 (7.68)	103 (22.58)	121(26.54)	144(31.58)	53(11.62)	0.45	**< 0.001**	0.047	**0.012**	0.43	**0.046**	0.31	0.231
CAMs offer patients cost-effective treatment options	33 (7.24)	75 (16.45)	141(30.92)	168(36.84)	39(8.55)	0.23	**0.002**	0.284	0.71	0.19	0.053	**0.049**	0.81
CAMs need more hospital-based research	27 (5.92)	79 (17.33)	107(23.46)	168(36.84)	75(16.45)	0.78	**0.04**	0.731	0.213	0.79	0.059	0.21	0.751
CAMs need more scientific testing before being used in conventional medicine.	28 (6.14)	74 (16.22)	113(24.78)	178(39.04)	63(13.82)	0.63	**0.049**	0.51	**0.041**	0.21	**0.02**	**0.04**	0.32
CAMs have a more holistic approach to health than conventional medicine.	25 (5.48)	97 (21.27)	165(36.19)	156(34.21)	13(2.85)	0.19	**0.016**	0.27	0.78	0.171	**0.03**	**0.010**	0.09

SD strongly disagree, D Disagree, N Neutral, A Agree, SA Strongly agree. A: age, P: Profession, G: Gender, AP: area of practice, CP: category of Practice, MS: Marital Status, R: Region. Note: Bold values show a significant association *test applied: chi score

### Perceptions of CAM modalities

Questions about the effectiveness and harmfulness of five different CAM modalities were asked from participants. Table 3 shows that the majority of participants considered these CAM modalities effective. The number of participants who considered spiritual healing and herbal therapies effective is greater than the participants who considered other therapies effective. Herbal therapies were revealed to have a significant association with profession, and experience of practice (p=0.001 &0.02) Almost 65% of participants considered spiritual healing effective with significant differences by age, profession, gender, experience of practice, and marital status (p = 0.02, 0.02, 0.02, 0.04 & 0.02 respectively) 57% of participating healthcare professionals reported Acupuncture therapy as effective therapy with a significant difference among professionals. Almost 49% of participants gave a vote for the effectiveness of Chinese medicine which was less than votes for the effectiveness of other CAM modalities with a significant difference by professionals (p = 0.0.02) Spiritual healing was considered least harmful in all CAM modalities while homeopathic medicine was considered more harmful than other four described type of CAM. The associations of perception towards different CAM modalities with demographics are presented in Table 3.

**Table 3:** Perception of HCPs toward CAM Modalities and difference in responses by demographic characteristics

Question about perception	VE	E	NI	H	VH	A	P	G	EP	AL	CP	MS	R
Herbal therapy (Effective or harmful)	99(21.71)	255(55.93)	73(16.00)	14(3.07)	15(3.29)	0.21	**0.001**	0.006	**0.02**	0.49	0.19	0.19	0.21
Homeopathic medicine (effective or harmful)	56(12.28)	204(44.74)	111(24.34)	74(16.23)	11(2.41)	0.099	0.05	**0.01**	**0.013**	0.37	**0.02**	0.79	0.52
Spiritual healing (effective or harmful)	115(25.22)	184(40.35)	132(28.95)	16(3.51)	9(1.97)	**0.02**	**0.02**	**0.02**	**0.04**	0.49	0.10	**0.02**	0.31
Acupuncture therapy (effective or harmful)	74(16.23)	188(41.22)	140(30.70)	34(7.46)	20(4.39)	0.49	**0.01**	0.49	0.22	0.27	0.25	0.22	0.21
Chinese medicine (effective or harmful)	42(9.21)	181(39.69)	168(36.85)	37(8.11)	28(6.14)	0.19	**0.02**	0.31	0.28	0.19	0.251	0.171	0.20

VE = very effective, E = effective, NI = no idea, H = harmful, VH = very harmful Note: Bold values shown significant association

### Integration of CAM

The purpose of this section was to assess the attitudeof HCPs toward the integration of CAM education intothe curriculum of undergraduate health programs. As shown in Table 4, for different items responses of participants were different. The majority of participants opposed the statement ‘’introduction of CAM topic will lengthen the study period’ ’with a significant difference by profession (p = 0.042), the experience of practice (p = 0.01), and category of experience (p = 0.02). But on another hand, the majority of participants showed a positive attitude toward the statement ‘’CAM knowledge is necessary for health care professionals’’ with a significance difference by age (p = 0.041), profession (p = 0.02), and experience of practice (p = 0.01). Almost 40% of participants thought that CAM course was required for health care professionals with a significant difference by profession (p = 0.01), the experience of practice (p = 0.02), and category of practice (p = 0.02). Similarly, 37% of participants disagreed that showed CAM courses should be offered at the postgraduate level. This response was significantly associated among categories of practice (p = 0.001). Almost 53% of participants believed that CAM could be offered as an elective course and on another hand, 51% of participants opposed that CAM should be made compulsory in primary degrees with a significant difference among professions (p = 0.01) Results of this section indicates that HCPs wants CAM course and CAM knowledge but they want CAM course in their curriculum as an elective course, not as a compulsory course.

**Table 4:** Opinion of HCPs about CAM education integration in curriculum and difference inresponses

Questions about integration of CAM	SA	A	N	D	SD	A	P	G	EP	AL	CP	MS	R
CAM knowledge is necessary for health professionals	88(19.30)	222(48.68)	96(21.05)	35(7.68)	15(3.29)	**0.041**	**0.02**	0.927	**0.01**	0.28	0.61	0.25	0.21
CAM should be compulsory in the primary degree	16(3.51)	79(17.32)	128(28.07)	176(38.60)	57(12.5)	0.21	**0.001**	0.504	0.24	0.32	0.21	0.19	0.91
CAM can be offered as an elective course	34(7.46)	208(45.61)	131(28.73)	65(14.25)	18(3.95)	0.18	**0.021**	0.231	0.182	0.61	0.42	0.31	0.28
CAM courses should be offered at the postgraduate level	33(7.24)	98(21.49)	148(32.45)	141(30.92)	36(7.90)	0.39	0.51	0.098	0.49	0.71	**0.001**	**0.02**	0.51
The introduction of the CAM topic will lengthen the study period	27(5.92)	85(18.64)	131(28.73)	155(33.99)	58(12.72)	0.681	**0.042**	0.48	**0.01**	0.17	**0.02**	0.61	0.29
CAM course is not required for health professionals	39(8.55)	106(23.25)	127(27.85)	148(32.46)	36(7.89)	0.131	**0.001**	0.191	**0.02**	0.32	**0.02**	0.31	0.119

SA Strongly agree, A Agree, N Neutral, D Disagree, SD strongly disagree, note: Bold values show a significant association

### Knowledge about CAM

Almost more than 50% of the participants gave incorrect responces to all questions except three questions (Q#3, 5 & 12); it meant that the majority of HCPs were aware of common uses of garlic, sharbat tootsiyah, and acupuncture therapy in Pakistan which were used for raised cholesterol, respiratory problems, and pain management respectively. Almost 15% of participants were aware of the use of chicorium seeds and chlorphytumborivilianum which are used for liver and sexual problems respectively. The majority of healthcare professionals gave incorrect answers to questions about the uses of St John’s wort, ginkgo biloba, and sharbat Bazori which are used for depression, mental problems, and liver ailments respectively. Likewise, most of the participants were unaware of highly diluted preparations of homeopathy and the concept of essential body fluid in unani medicine. Similarly, 63% of participants were unaware of the fact ‘’in Pakistan people visit spiritual healers mostly for the treatment of magic’’. Khameeragauzban (Borago officinalis L.) is a very famous herbal product that is used for anxiety. Only 29% HCPs were aware ofthe indication of khameeragauzban. The majority of respondents were not familiar with the uses of two famous homeopathy medicine; Berberis vulgaris and Ignatia which are used for kidney stones and anxiety. Percentages of all correct and incorrect responses against each statement about knowledge are given in Table 5. Overall, 361 (79.17%) HCPs have poor knowledge and 95 (20.83%) HCPs have good knowledge about CAM. The result revealed that physicians have the highest knowledge score (25.63%) among professionals, followed by pharmacists (21.99%) and nurses (12.26%) As shown in Table 6, knowledge status was significantly associated with age, profession, and experience of practice (p = 0.001, 0.001, 0.019 respectively).

**Table 5:** Correct and incorrect answers by healthcare professionals

Sr. No.	Questions	Correct n (%)	Incorrect n (%)
**1.**	St. John’s Wort is commonly used for the Treatment of	151(33.11)	305 (66.89)
**2.**	Ginkgo biloba is commonly used in people with ------- disease	137 (30.04)	319 (69.96)
**3.**	Garlic can lower -------- levels to a mild extent	228 (50)	228 (50)
**4.**	------- is a form of alternative medicine in which practitioners use highly diluted preparations	165 (36.18)	291 (63.82)
**5.**	A major use of Acupuncture in Pakistan is for ------	278 (60.96)	178 (39.04)
**6.**	Ignatia (St. Ignatius bean) in homeopathy is used	133 (29.17)	323(70.83)
**7.**	Peoples visit Spiritual healers mostly for the treatment of -----	169 (37.06)	287 (62.94)
**8.**	------- medicines system works on the principle of “reinforcing the natural defense of the body “	119 (26.10)	337 (73.90)
**9.**	According to ------- medicine system, an imbalance of essential body fluid causes diseases	87(19.08)	369 (80.92)
**10.**	Liquorice ( mulathi ) is used	201 (44.08)	255 (55.92)
**11.**	Chlorophytum borivilianum (muslisafaid) is mostly used for----diseases.	68 (14.91)	388 (85.09)
**12.**	Sharbat tootsiyah is used for -------- problems	24 (53.07)	214 (46.93)
**13.**	Chicorium seeds (kasni seeds) are mostly used for ------ diseases.	65 (14.25)	391 (85.75)
**14.**	Khameeragaozban is used for …….	133 (29.17)	323 (70.83)
**15.**	Sharbat e bazori is indicated in sluggish liver & renal calculus, It is	210 (46.05)	246 (53.95)
**16.**	Berberis Vulgaris & Cantharis are famous homeopathy medicines, are used for	119 (26.10)	337 (73.90)

**Table 6:** Knowledge and difference in responses by demographic characteristics

Variable	Good Knowledge n (%)	Poor Knowledge n (%)	P-value
	95 (20.83)	361 (79.17)	
Age			
20–30	54 (18.43)	239(81.57)	
31–40	24 (20)	96 (80)	**0.001**
Above 40	11(25.58)	32(74.42)	(significance)
**Profession**			
Physician	41 (25.63)	119 (74.37)	
Pharmacist	31 (21.99)	110(78.01)	**0.001**
Nurse	19 (12.26)	136(87.74)	(significance)
**Gender**			
Female	52 (16.88)	256 (83.12)	0.119
Male	37 (25)	111 (75)	(non-significance)
**Years of experience**			
0–3	38 (13.97)	234 (86.03)	
4–6	25(24.27)	78 (75.73)	**0.019**
7–9	19(35.85)	34(64.15)	(significance)
Above 9	6(21.43)	22 (78.57)	
**Area of living**			
Rural	40 (29.85)	94(70.15)	0.682
Urban	65(20.19)	257(79.81)	(non-significance)
**Category**			
Secondary	53(25.12)	158(74.88)	0.070
Tertiary	63 (25.71)	182 (74.29)	(non-significance)
**Marital Status**			
Married	42 (23.33)	138 (76.67)	0.230
Unmarried	48(17.39)	228 (82.61)	(non-significance)
**City**			
Islamabad	46(16.55)	232 (83.45)	0.073
Rawalpindi	46(25.84)	132(74.16)	(non-significance)

Spearman correlation tests demonstrate a strong linear positive correlation between attitude and perception (r = 0.550, p < 0.001). However, there is a weak positive correlation between knowledge-attitude (r = 0.090, p = 0.04). Similarly, a weak positive correlation exists between attitude and opinions about integration (r = 0.099, p = 0.039).

## Discussion

HCPs should have adequate knowledge of CAM products for effective patient recommendations. To the best of our knowledge,this is the first study conducted in Pakistan that has explored the KAP of Pakistani HCPs towards CAM. Our study demonstrates that majority of participants perceive CAM therapies as an effective treatment option but have poor knowledge of such therapies. The findings of this survey are comparable to some other studies conducted in Iran [20, 37] and India [38, 39]. According to this study, the majority of participants reported that CAM includes the ideas and methods from which conventional medicine could benefit reflecting a positive attitude towards effective CAM modalities are consistent with study conducted in Ethiopia [40]. Similarly, most of the HCPs perceived different CAM modalities as effective. These findings are consistent with other studies conducted in Ethiopia [16] and India [38] Almost 80% of the participants had poor knowledge about CAM. This knowledge score aligns with the study conducted in Iraq and Iran [13, 35]but is lower than the studies conducted in Ethiopia [16] and Lebanon [24].

Most of the participants (42.32%) opposed the integration of CAM and conventional medicine practices for clinical care. This response is contradicted to findings of an Ethiopian study, where more than half of the participating HCPs favored integrating conventional and CAM methods [16]. The possible difference for this might be due to the different interests of HCPs in CAM products, the availability of registered products of CAM, and the regulatory rules of countries. More than half of HCPs suggested that knowledge of HCPs about CAM, hospital-based research, and scientific testing of CAM products is vital before the use in a streamlined medicine system, which is compatible withstudies conducted in Palestine [22] and Guyana [41]. 41.88% of HCPs participating in this study agreed that CAM modalities include ideas and methods from which conventional medicine systems could benefit. This result was harmonious with findings reported in a study conducted in Guyana [41]. Most of the HCPs participating in this study agreed that CAMs offer patients cost-effective treatment options. This response is congruent to the study conducted in Ethiopia, in which 63.4% of the participants felt that CAM offers cost-effective treatment [16]. Almost half of the participants thought that health and disease are a balance between enhancing and destructive forces, which is compatible with a study conducted in Guyana. It seems that the overall positive attitude of the participating HCPs towards CAM modalities is due to cost-effectiveness, natural origin, and traditional factors.

The number of participants who considered spiritual healing and herbal therapies as effective treatment options is greater than the participants who considered other CAM therapies effective. However, a higher number (up to 77%) of HCPs participating in this study perceived herbal therapy, Homeopathy, and Acupuncture to be effective compared to the report by general physicians in a study of Islamabad [42] where only up to 50% of physicians perceived these therapies as effective. This difference could be due to the different types of healthcare professionals in this study, as perceptions of the effectiveness of these CAM therapies were significantly varied among professionals. On other hand, observed findings on the perceptions about herbal, homeopathy, acupuncture, and spiritual healing agree with the Indian study [38]. These compatible results in two neighboring countries could be due to the similar cultural norms, and availability of educational institutions for CAM practitioners. Almost 49% of participants voted for the effectiveness of Chinese medicine which was less than the votes for the effectiveness of other CAM modalities with significant differences by professionals. The possible reason could be the lesser availability of Chinese medicine products in Pakistan. Spiritual healing perceived least harmful in all CAM therapies may be associated with the religious beliefs of participants and their families. On the other hand, homeopathic medicine was considered more harmful among all five types of CAM Modalities mentioned. This type of perception could be due to the low knowledge of HCPs about this therapy.

The majority of the participants agreed that CAM knowledge and CAM courses are necessary for HCPs of conventional medicine. Similarly, more than half of the participating HCPs thought that CAM could be offered as an elective course; these findings are consistent with studies conducted inAustralia [43], India [38], Trinidad and Tobago [44]. On other hand, 51% of participants opposed that CAM should be made compulsory in the primary degree. Findings of all these six statements about integrating CAM education in the curricula of HCPs courses indicate that HCPs suggest CAM courses as elective subjects, not as compulsory. The length of medical science courses and the tough external evaluation system of Pakistani medical institutions could be reasons behind this type of response.

The reported poor knowledge score (20.83%) is compatible with studies carried out in Iraq [45], Iran [20, 37], and Trinidad and Tobago [44]. On the other hand, reported knowledge scores of Pakistani HCPs are lower than the studies done in Lebanon [24], Palestine [22], Italy [3], South Ethiopia [16], and Australia [46]. The reason for the low knowledge score might be the lack of training and outdated syllabi of undergraduate health sciences programs. Although the knowledge scores of HCPs about different CAM products and concepts were low, in general, most of them were aware of the uses of herbal products and acupuncture techniques. After the commencement of Alternative Medicines and Health Products (Enlistment) Rules, 2014 homeopathic and herbal products are freely available in community pharmacies therefore HCPs of Pakistan must have basic knowledge aboutthe indication, dose, contraindication, and side effects of some commonly used alternative products to counsel the patients [19]. Physicians have the highest knowledge score among HCPs followed by pharmacists and nurses, compatible with the findings of the study conducted Trinidad and Tobago, where the knowledge score of physicians was comparatively higheramong other professionals [44]. Study material about CAM products often received by physiciansfrommanufacturer representatives might be the reason for a higher score of physicians.

Poor knowledge scores and a positive attitude towards CAM reflect the need to update the health sciences undergraduate curricula and involve HCPs in training programs and optional courses. As CAM products are included in the therapeutic goods list by DRAP, advisories about these products should be published for HCPs. For the evidence-based practice of CAM in the future, relevant councils should upgrade the syllabi of educational programs about CAM and also offer short courses for HCPs of conventional medicine systems.

### Strengths and limitations

The strength of this research is the reality that it is the first survey that included data about the KAP of physicians, pharmacists, and nurses towards CAM in a representative study of the Pakistani population. Secondly, a sufficiently representative sample size for the selected population is another strength of this study. Our research has some limitations: (I) for statistical interpretation, the “no idea” answers were included in the number of wrong responses on CAM knowledge assessment. Participants were guided to choose “no idea” instead of inappropriately estimating an answer. This may have declined the overall score in assessing the HCPs’ Knowledge of CAM. (II) The possibility of wrong judgment by participants must be considered, therefore it was presumed that every participant translates each statement in its intended form. (iii) level of education of different healthcare professionals was not assessed, instead level of experience was assessed.

## Conclusion

This survey revealed that most of HCPs had positive attitudes and perceptions toward different CAM therapies. The majority of the HCPs showed interest in scientific testing of CAM products and integration of CAM education in the curriculum of their respective primary degree as elective courses. Despite having a welcoming attitude, the knowledge score of HCPs about CAM was low. There is a dire need to arrange training programs on CAM and improve the existing curriculum to bridge the knowledge gap and promote better health outcomes.

## Data Availability

The data supporting this study’s findings are available from the corresponding author on a reasonable request.
